# An adjustment in BMP4 function represents a treatment for diabetic nephropathy and podocyte injury

**DOI:** 10.1038/s41598-018-31464-9

**Published:** 2018-08-29

**Authors:** Yui Fujita, Tatsuya Tominaga, Hideharu Abe, Yumi Kangawa, Naoshi Fukushima, Otoya Ueda, Kou-ichi Jishage, Seiji Kishi, Taichi Murakami, Yumiko Saga, Yashpal S. Kanwar, Kojiro Nagai, Toshio Doi

**Affiliations:** 1 Department of Nephrology, Graduate School of Biomedical Science, Tokushima University, Tokushima, Japan; 2 Research Division, Fuji Gotemba Research Labs, Chugai Pharmaceutical Co., Ltd, Shizuoka, Japan; 3 Chugai Research Institute for Medical Science Inc., Shizuoka, Japan; 4 Division of Mammalian Development, Genetic Strains Research Center, National Institute of Genetics, Mishima, Shizuoka, Japan; 5 Department of Pathology & Medicine-Nephrology, FSM, Northwestern University, Chicago, Illinois 60611 USA

## Abstract

Podocyte injury has been proposed to play an important role in diabetic nephropathy; however, its pathological mechanism remains unclear. We have shown that bone morphogenetic protein 4 (BMP4) signaling leads to the glomerular changes characteristic of this disorder. To analyze the molecular mechanism of podocyte injury, the effect of BMP4 was investigated using streptozotocin (STZ)-induced, *Bmp4* heterozygous knockout (*Bmp4*+/−) and podocyte-specific *Bmp4* knockout mice. Mice with STZ-induced diabetes exhibited glomerular matrix hyperplasia and decreased numbers of podocyte nucleus-specific WT1-positive cells. The number of podocytes and proteinuria were improved in both diabetic *Bmp4* knockout mouse models compared to the effects observed in the control mice. The effect of BMP4 overexpression on *Bmp4*-induced or podocyte-specific transgenic mice was examined. Tamoxifen-induced *Bmp4*-overexpressing mice exhibited mesangial matrix expansion and decreased numbers of WT1-positive cells. Podocyte-specific *Bmp4*-overexpressing mice displayed increased kidney BMP4 expression and mesangial matrix expansion but decreased nephrin expression and numbers of WT1-positive cells. Both lines of *Bmp4*-overexpressing mice exhibited increased albuminuria. In cultured podocytes, BMP4 increased phospho-p38 levels. BMP4 decreased nephrin expression but increased cleaved caspase-3 levels. p38 suppression inhibited caspase-3 activation. Apoptosis was confirmed in STZ-diabetic glomeruli and *Bmp4*-overexpressing mice. *Bmp4* +/− mice with diabetes displayed reduced apoptosis. Based on these data, the BMP4 signaling pathway plays important roles in the development of both podocyte injury and mesangial matrix expansion in diabetic nephropathy.

## Introduction

The global prevalence of diabetic nephropathy (DN) has dramatically increased among patients with chronic kidney disease. The associated kidney damage has been shown to be related to functional and structural changes in glomerular cells, including mesangial cells, podocytes, and endothelial cells^1–3^. Additionally, several recent studies have focused on examining the cellular interactions between different glomerular cells. In particular, mesangial cells are the main target for the development of characteristic mesangial matrix expansion in subjects with DN^4,5^. As shown in our previous study, bone morphogenetic protein 4 (BMP4)/Smad1 signaling plays an important role in the development of mesangial cell injury in DN^6,7^. Tamoxifen-inducible *Bmp4* transgenic mice (tgm), which primarily express BMP4 in glomerular cells, showed dramatic mesangial matrix expansion comparable to that of subjects with DN. However, the involvement of specific cell types and their role(s) in the pathological reaction remain unknown *in vivo*.

Podocytes are terminally differentiated cells that play an important role in maintaining the barrier function and glomerular structure required to prevent protein leakage into the urine^8^. Proteinuria has been shown to induce glomerular injury by promoting the loss and dysfunction of podocytes^9,10^. Apoptosis constitutes one of the main pathways contributing to podocyte loss, which leads to glomerulosclerosis^11^. Podocytes are subjected to various stresses, including oxidative stress, endoplasmic reticulum stress, metabolic stress, inflammation, toxins, and physical-mechanical stimuli^12,13^. These factors may be involved in the complex mechanism underlying the development of kidney damage. For example, high glucose and advanced glycated proteins induce podocyte apoptosis, a process mediated by transforming growth factor beta (TGFβ), in conjunction with p38^14^. Specifically, the activation of the p38 MAP kinase and caspase-3 in cells exposed to stress or cytokines triggers the apoptotic response in podocytes.

BMPs are expressed during kidney development and affect tissue morphogenesis. BMP4 is an important molecule that induces the stem cell differentiation required for the development of various organs, including the kidney^15–17^. BMP4 also induces Smad1/5/8 phosphorylation and p38 activation during embryonic tissue formation, triggering apoptosis^18^. Additionally, the BMP4-Smad1 pathway mediates senescence in injured cells^19^. Consistent with podocyte injury in nephrotic syndrome, which is caused by apoptosis and cell cycle dysfunction, we have reported that BMP4 constitutes a critical molecule that induces glomerulosclerosis in subjects with DN. In addition, the expression of p16 and p21 in response to BMP4 has been shown to be mediated by the Smad1/5/8 signaling pathway^20^. Smad1 upregulates the transcription of type IV collagen, the primary protein component of the extracellular matrix in mesangial cells^6^. Notably, mesangial matrix hyperplasia in DN mice is suppressed by a BMP4 neutralizing antibody^21^. Thus, BMP4/Smad1 signaling plays a central role in the development of mesangial matrix hyperplasia in subjects with DN; however, the precise molecular mechanisms by which BMP4 induces podocyte injury remain unclear. In this study, we used *Bmp4* heterozygous knockout mice (*Bmp4*+/−), podocyte-specific *Bmp4* knockout mice, induced *Bmp4* tgm, and podocyte-specific *Bmp4* tgm to examine the molecular mechanism by which the BMP4/Smad1/p38 signaling pathway mediates glomerular injury in a mouse model of DN.

## Results

### Diabetic Bmp4 heterozygous knockout mice exhibit reduced podocyte injury

**Figure 1:**
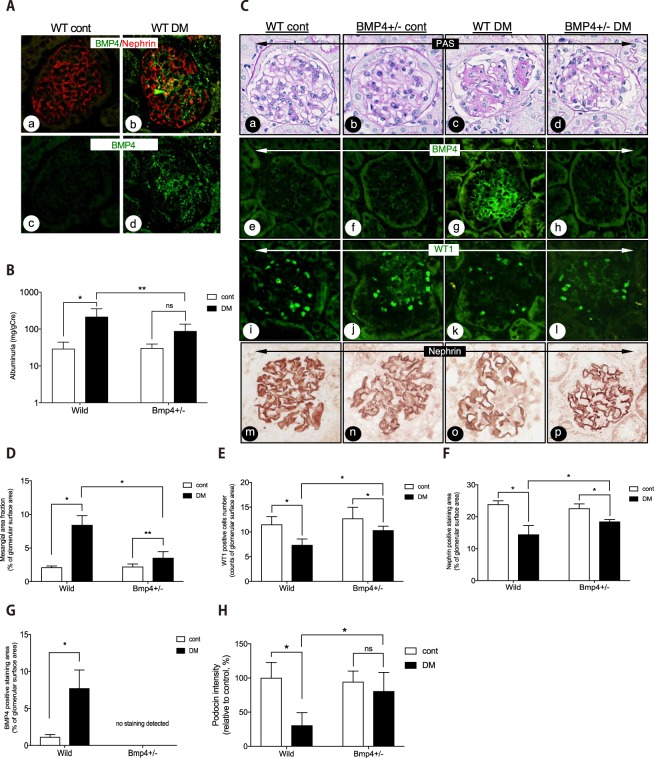
Diabetic Bmp4 heterozygous knockout mice (Bmp4+/−) exhibited reduced podocyte injury and mesangial matrix expansion. (**A**) Images of BMP4 and nephrin immunohistochemistry in the glomeruli. In diabetic C57BL/6 mice (WT DM) (b), BMP4 expression was increased in the glomerulus compared with the control mice (WT cont) (a). The BMP4-expressing areas are depicted in green, and the nephrin-expressing area is shown in red. Yellow indicates the area of BMP4 and nephrin overlap (b, arrowhead). **(B)** Albuminuria was 214.7 mg/g Cre for WT DM mice and 87.9 mg/g Cre for *Bmp4* +/− DM mice. A significant difference was not observed between Bmp4 +/− cont and Bmp4 +/− DM mice. **(C)** Representative images of light microscopy and immunohistochemistry in the glomeruli. WT DM mice exhibited increased BMP4 expression (g) and decreased numbers of WT1-positive cells (k) and nephrin-expressing areas (o) compared with the WT controls. *Bmp4* +/− DM mice did not display BMP4-positive staining (h). *Bmp4* +/− DM mice exhibited a moderate decrease in the number of WT1-positive cells (l) and nephrin expression (p) compared with WT DM mice. (**D)** The mesangial area fraction was determined by measuring the PAM-stained area. A significant difference was not observed between WT cont and *Bmp4* +/− cont mice. WT DM displayed mesangial matrix expansion compared with the control mice. *Bmp4* +/− DM mice exhibited decreased mesangial matrix expansion compared with WT DM mice. **(E)** The number of WT1-positive cells was determined by immunostaining. A greater number of WT1-positive cells was observed in *Bmp4* +/− DM mice than in WT DM mice. **(F)** The nephrin-positive area was determined by measuring the immunostaining. A larger nephrin-positive area was observed in *Bmp4* +/− DM mice than in WT DM mice. **(G)** The BMP4-positive area was determined by measuring the immunostaining. WT DM mice exhibited increased BMP4 expression. **(H)** Podocin levels were determined by western blotting. Levels in WT DM mice were 30.5% and levels in *Bmp4* +/− DM mice were 80.7% compared to WT cont mice. Cont, nondiabetic mice; DM, diabetic mice. ^*^P < 0.01, ^**^P < 0.05 (ANOVA).

We first examined the development of DN in STZ-induced diabetes models using *Bmp4* +/− mice. High blood glucose levels were observed at 24 weeks after the induction of diabetes in either wild-type or *Bmp4* +/− mice, as we have previously reported^7^. Glycoalbumin levels were also significantly increased in the diabetic group (Supplemental Table 1A). A significant difference in blood glucose levels was not observed between the wild-type and *Bmp4* +/− *mice*. The diabetic mice exhibited significantly increased mesangial matrix expansion and BMP4 levels compared to those in the control mice (Fig. 1C-a and c). The area of BMP4 expression partially overlapped podocytes, but BMP4 expression was also observed elsewhere, including primarily mesangial areas (Fig. 1A-b).

Next, we investigated whether a decrease in BMP4 expression attenuated any renal changes in diabetic mice. Mesangial matrix expansion, BMP4 expression, and albuminuria were attenuated in the diabetic *Bmp4* +/− mice compared with the diabetic wild-type mice (Fig. 1B,C-g-h and D). The results of periodic acid methenamine silver (PAM)-stained areas in the wild-type mice were compatible with findings from our previous report^7^. A statistically significant difference in the albuminuria levels was not observed between the diabetic and non-diabetic *Bmp4* +/− mice. The podocyte number, as determined by the number of WT1-positive cells, was maintained in the diabetic *Bmp4* +/− mice compared to the diabetic wild-type mice (Fig. 1C-k,l and E). Furthermore, the diabetic *Bmp4* +/− mice expressed higher levels of nephrin than the diabetic wild-type mice (Fig. 1C-o,p and F). BMP4-positive staining was only observed in the wild-type mice, and the area was increased in the diabetic animals (Fig. 1G). Western blot analyses revealed decreased expression of podocin in the diabetic mice. The diabetic *Bmp4* +/− mice exhibited similar levels of podocin as the nondiabetic mice (Fig. 1H). In addition, the creatinine clearance rate (Ccr) was slightly reduced in the diabetic *Bmp4* +/− mice (Supplemental Table 1A).

### Podocyte-specific Bmp4 knockout mice with diabetes exhibit reduced podocyte injury

**Figure 2:**
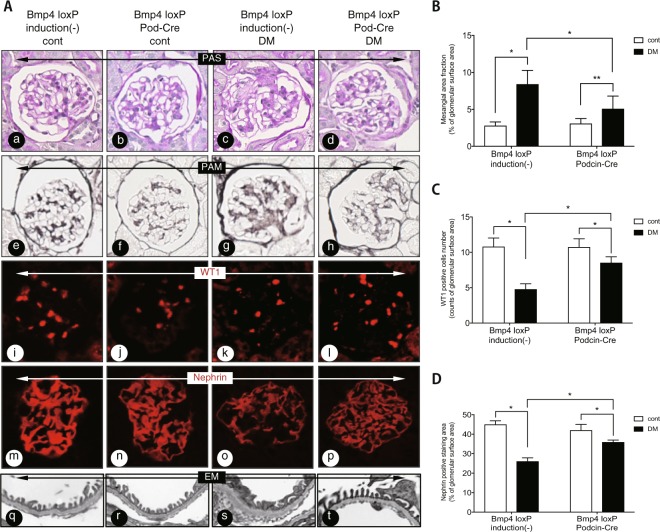
Diabetic podocyte-specific Bmp4 knockout mice exhibited reduced podocyte injury and mesangial expansion. **(A)** Representative light microscopy, immunohistochemistry and electron microscopy images of glomeruli. Extracellular matrix deposition was determined by PAS staining. PAS staining was increased in diabetic *Bmp4*^*loxP*^ induced (−) mice (c) and decreased in diabetic *Bmp4*^*loxP*^ × *Podocin*-Cre mice (d). Diabetic *Bmp4*^*loxP*^ × *Podocin*-Cre mice exhibited a moderate decrease in the number of WT1-positive cells (l) and nephrin expression (p) compared with diabetic *Bmp4*^*loxP*^ induced (−) mice. The GBM thickness in the diabetic *Bmp4*^*loxP*^ × *Podocin*-Cre mice was reduced compared with the diabetic *Bmp4*^*loxP*^ induced (−) mice (s and t). **(B)** A bar graph summarizes the histological scores for PAM staining. Diabetic *Bmp4*^*loxP*^ × *Podocin*-Cre mice displayed decreased mesangial matrix expansion compared with diabetic *Bmp4*^*loxP*^ induced (−) mice. **(C)** The number of WT1-positive cells was determined by immunostaining. A greater number of WT1-positive cells was observed in the diabetic *Bmp4*^*loxP*^ × *Podocin*-Cre mice than in the diabetic *Bmp4*^*loxP*^ induced (−) mice. **(D)** The nephrin-positive area was determined by measuring the immunostaining. A larger nephrin-positive area was observed in the diabetic *Bmp4*^*loxP*^ × *Podocin*-Cre mice than in the diabetic *Bmp4*^*loxP*^ induced (−) mice. Cont, nondiabetic mice; DM, diabetic mice. ^*^P < 0.01, ^**^P < 0.05 (ANOVA).

We investigated whether the podocyte-specific reduction in levels of secreted BMP4 attenuated any renal changes in diabetic mice. *Bmp4*^*loxP*^ × *Podocin*-Cre mice exhibited decreased *Bmp4* levels in the kidneys, as detected by quantitative PCR. *Bmp4*^*loxP*^ induction (−) in control mice has not been crossbred with *Podocin-*Cre mice. Mesangial matrix expansion was attenuated in the diabetic *Bmp4*^*loxP*^ × *Pod*-Cre mice compared with the diabetic *Bmp4*^*loxP*^ induced (−) mice (Fig. 2A-c-d and B). The reduction in WT1- and nephrin-positive areas was alleviated in these mice compared to the diabetic non-induced mice (Fig. 2 A-k-l,o-p,C,and D). Electronic microscopy analyses of diabetic *Bmp4*^*loxP*^ × *Pod*-Cre mice showed normal podocyte foot processes and glomerular basement membranes (GBMs) (Fig. 2A-t). Diabetic *Bmp4*^*loxP*^ induced (−) mice displayed marked alterations such as GBM thickening and podocyte foot process effacement (Fig. 2A-s). In addition, Ccr and albuminuria were not different between diabetic *Bmp4*^*loxP*^ × *Pod*-Cre mice and non-diabetic mice (Supplemental Table 1B).

### Bmp4 overexpression induces podocyte injury and mesangial matrix expansion *in vivo*

**Figure 3:**
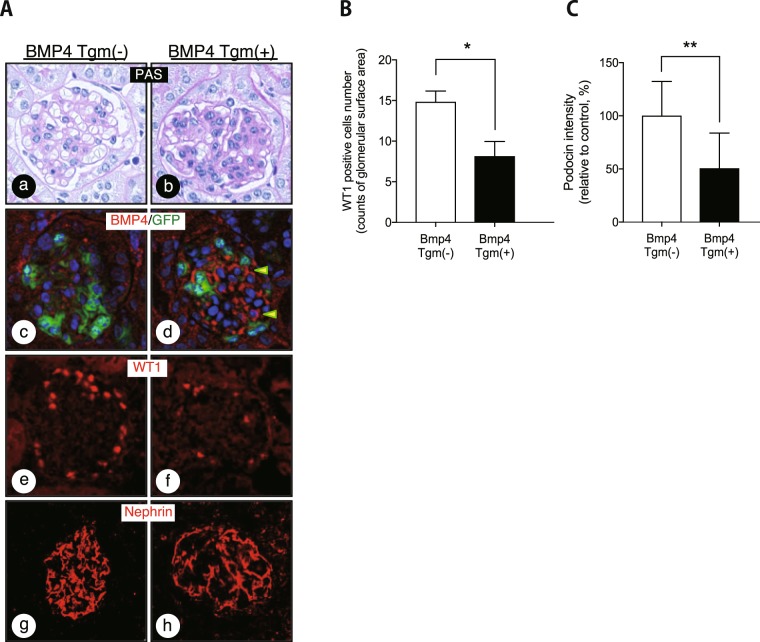
Tamoxifen-induced Bmp4 tgm exhibited podocyte injury and mesangial matrix expansion. **(A)** Representative light microscopy and immunohistochemistry images of glomeruli. Tamoxifen-induced expression of BMP4 in the transgenic mice resulted in the expansion of the mesangial area (b). Tamoxifen administration reduced GFP fluorescence and increased levels of the BMP4 protein. BMP4 was located in cells that previously expressed GFP (d). Compared to untreated animals, the WT1- (f) and nephrin-positive areas (h) were reduced in the tamoxifen-treated BMP4 tgm. **(B)** The number of WT1-positive cells was determined by immunostaining. Fewer WT1-positive cells were observed in *Bmp4* tgm (+) than in *Bmp4* tgm (−). **(C)** Podocin levels were determined by western blotting. Podocin levels were decreased in *Bmp4* tgm (+) compared with *Bmp4* tgm (−). ^*^P < 0.01, ^**^P < 0.05 (t-test).

To investigate the role of BMP4 in adult mice, we generated *Bmp4* tgm using the tamoxifen-regulated Cre-loxP system. As previously reported (ref.^7^). The *Bmp4* tgm (+) is a tamoxifen-induced BMP4-expressing adult mouse strain. *Bmp4* tgm (−) control mice were not induced with tamoxifen. *Bmp4* tgm (+) displayed increased BMP4 expression in whole glomeruli, as shown in red (Fig. 3A-d) and glomerulosclerosis (Fig. 3A-b). These mice exhibited a significant decrease in the number of WT1-positive cells and nephrin-positive area compared to *Bmp4* tgm (−) control mice (Fig. 3A-f and h). *Bmp4* tgm (+) exhibited a decreased number of WT1-positive cells compared with *Bmp4* tgm (−) (Fig. 3B). We confirmed the podocin levels using western blotting *in vivo*. *Bmp4* tgm (+) displayed decreased podocin levels compared with those in *Bmp4* tgm (−) (Fig. 3C).

### Podocyte-specific Bmp4 transgenic mice display podocyte loss

**Figure 4:**
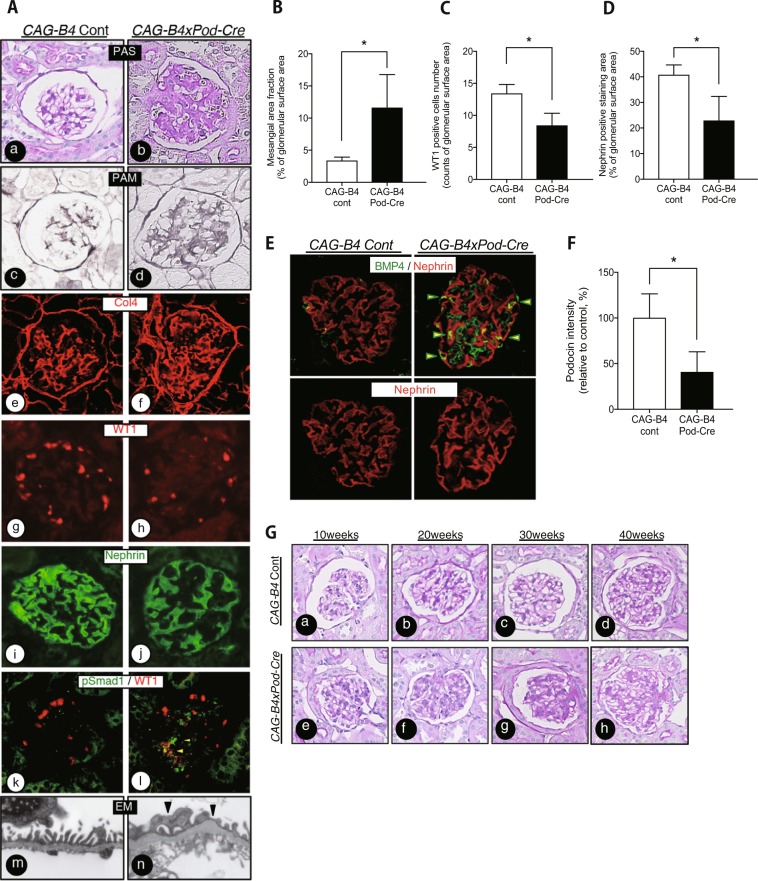
Podocin-Cre Bmp4 tgm exhibited glomerular injury. **(A)** Representative light microscopy, immunohistochemistry and electron microscopy images of glomeruli. The left panel shows a non-induced mouse (*CAG-Bmp4* cont), and the right panel shows a *Podocin*-Cre induced *Bmp4* tgm (*CAG-B4xPod-Cre*). Extracellular matrix deposition was determined by PAS staining. PAS staining was increased in *CAG-B4xPod-Cre* mice (b). The Col4-expressing area was increased in *CAG-B4xPod-Cre* mice (f). Compared to the *CAG-Bmp4* cont mice, the WT1- (h) and nephrin-positive areas (j) were reduced in *CAG-B4xPod-Cre* mice. The number of phospho-Smad1 (pSmad1)-positive cells was increased in *CAG-B4xPod-Cre* mice compared with *CAG-Bmp4* cont mice (l). The GBM thickness was increased in *CAG-B4xPod-Cre* mice compared with *CAG-Bmp4* cont mice (m and n). **(B)** A bar graph summarizes the histological scores of PAM staining. *CAG-B4xPod-Cre* mice exhibited mesangial matrix expansion compared with *CAG-Bmp4* cont mice. **(C)** The number of WT1-positive cells was obtained by immunostaining. Fewer WT1-positive cells were observed in *CAG-B4xPod-Cre* mice than in *CAG-Bmp4* cont mice. **(D)** The nephrin-positive area was determined by measuring the immunostaining. The nephrin-positive area was decreased in *CAG-B4xPod-Cre* mice compared with *CAG-Bmp4* cont mice. **(E)** Images of BMP4 and nephrin immunohistochemical staining in glomeruli. In *CAG-B4xPod-Cre* mice, BMP4 expression was increased in the glomerulus compared with that in *CAG-Bmp4* cont mice. The BMP4-expressing areas are shown in green, and the nephrin area is shown in red. Yellow indicates the area of BMP4 and nephrin overlap (arrowhead). **(F)** Podocin levels were determined by western blotting. Podocin levels were decreased in *CAG-B4xPod-Cre* mice compared with those in *CAG-Bmp4* cont mice. G) Glomerular PAS staining in each mouse from 10 weeks to 40 weeks of age. PAS staining was increased in *CAG-B4xPod-Cre* mice aged more than 30 weeks (g and h). ^*^P < 0.01, (t-test).

*CAG-B4* × *Pod-Cre* mice exhibited increased BMP4 expression in the kidney, as detected by western blotting and quantitative PCR. The *CAG-B4* control mice were not crossbred with *Podocin*-Cre mice. *CAG-B4* × *Pod-Cre* mice showed mesangial matrix hyperplasia at 30 weeks, and progressive mesangial matrix hyperplasia at 40 weeks of age (Fig. 4G-g and h). The 60-week-old mice developed severe mesangial sclerosis accompanied by interstitial fibrosis. In particular, the *CAG-B4* × *Pod-Cre* mice showed increased BMP4 expression in the glomerulus. These BMP4-expressing areas colocalized with podocytes, although staining was also observed in the area expressing collagen IV (Fig. 4E-b). These mice displayed fewer podocytes at 40 weeks of age than the reference mice (Fig. 4C), and they also exhibited significantly reduced nephrin expression (Fig. 4D). Furthermore, an increased number of pSmad1-positive cells was observed not only in partially WT1-positive cell areas but also among areas of predominantly mesangial cells (Fig. 4A-l). The electron microscopy analyses of these mice revealed GBM thickening, podocyte foot process effacement, and mesangial expansion, similar to the findings of DN mice (Fig. 4A-n). The *CAG-B4* × *Pod-Cre* mice exhibited decreased podocin levels compared with those in the *CAG-B4* control mice (Fig. 4F). In addition, the Ccr was decreased in the *CAG-B4* × *Pod-Cre* mice compared with the level in the *CAG-B4* cont mice. Albuminuria was increased in the *CAG-B4* × *Pod-Cre* mice compared with the level in the *CAG-B4* control mice. Systolic blood pressure levels were not different between the *CAG-B4* × *Pod-Cre* mice and the *CAG-B4* control mice (Supplemental Table 1D).

### Podocyte apoptosis is induced by the BMP4/p38 pathway

**Figure 5:**
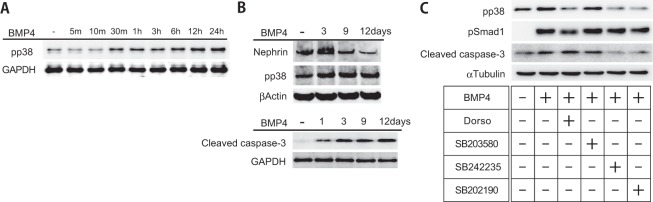
BMP4 induced apoptotic signaling in cultured podocytes. **(A)** Cultured podocytes were treated with BMP4 (20 ng/ml) at the indicated time points. BMP4 treatment of podocytes increased levels of phosphorylated p38 in a time-dependent manner. **(B)** BMP4 decreased nephrin expression and increased cleaved caspase-3 levels after 12 days of stimulation. **(C)** Cultured podocytes were untreated or incubated with dorsomorphin (Dorso), SB203580, SB242235 or SB202190 for 24 hr and then stimulated with BMP4. Each inhibitor was added to the culture medium at a concentration of 500 nM. The pp38 levels were decreased in cells treated with dorsomorphin, SB242235 and SB202190. The pSmad1 levels were decreased in response to dorsomorphin treatments. The cleaved caspase-3 levels were decreased in cells treated with SB242235 and SB202190. Total cell lysates were immunoblotted with anti-pp38, anti-nephrin, anti-cleaved caspase-3, anti-pSmad1, anti-GAPDH, anti-β-actin and anti-α-tubulin antibodies.

We studied cultured podocytes *in vitro* to investigate the mechanism by which BMP4 mediates podocyte injury. Podocytes were treated with BMP4 for 5 min to 24 h. Western blot analyses revealed a time-dependent increase in phosphorylated p38 levels following treatment with BMP4 (Fig. 5A). In contrast, nephrin expression was reduced by BMP4 (Fig. 5B). The BMP4 treatment induced caspase-3 cleavage in podocytes (Fig. 5B). P38 inhibitors (SB242235 and SB202190) inhibited the activation of p38 and cleaved caspase-3 (Fig. 5C). Conversely, dorsomorphin specifically inhibited the activation of Smad1, whereas cleaved caspase-3 levels were not affected (Fig. 5C).

### Changes in the levels of cleaved caspase-3 and apoptosis *in vivo*

**Figure 6:**
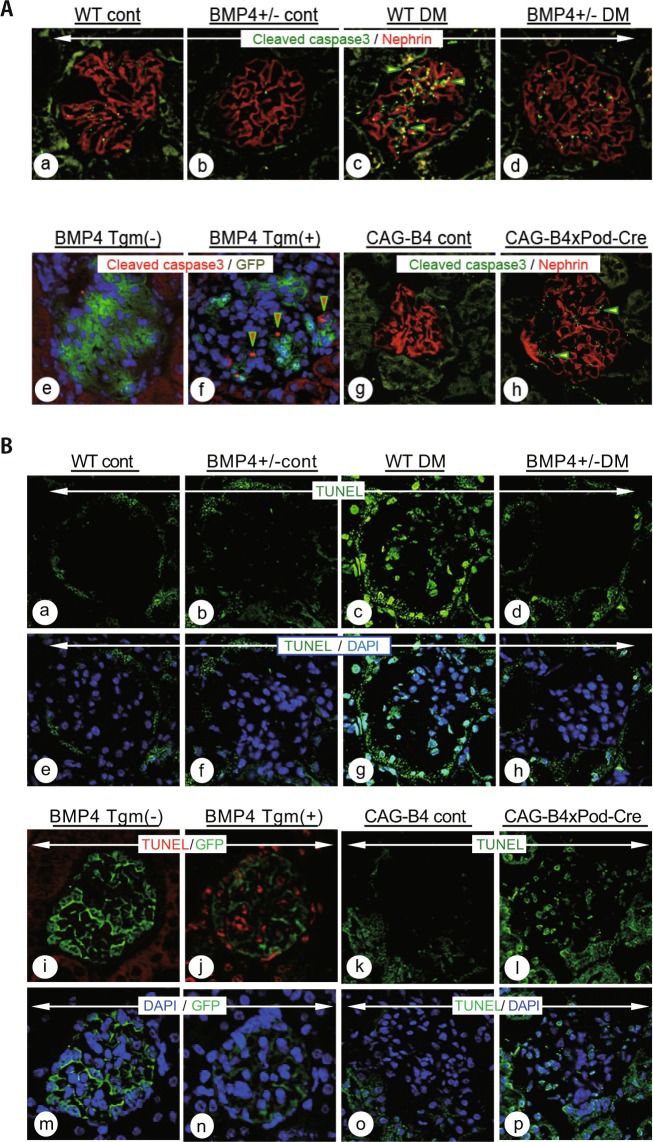
Diabetic mice and BMP4 induced mice exhibited apoptotic damage in glomeruli. **(A)** Images of immunohistochemistry for cleaved caspase-3 in the DM model, *Bmp4* tgm and *CAG-B4xPod-Cre* mice. The WT DM mice (c) exhibited an increase in the positively stained area compared with the WT control mice (a). Yellow represents the area in which cleaved caspase-3 and nephrin expression overlap (c, arrowhead). *Bmp4* +/− DM mice (d) exhibited a reduced positively stained area compared with WT DM mice. *Bmp4* tgm (+) (f) and *CAG-B4xPod-Cre* (h) mice displayed increased positively stained areas after Cre-mediated recombination. Cleaved caspase-3-stained areas are shown in red in *Bmp4* tgm (+) (f, arrowhead) and green in *CAG-B4xPod-Cre* mice (h, arrowhead). **(B)** Percentage of apoptotic cells in the glomeruli, as determined by a TUNEL assay. Apoptosis was frequently observed in the glomeruli of WT DM (c), *Bmp4* tgm (+) (j, red color) and *CAG-B4xPod-Cre* mice (l). *Bmp4* +/− DM mice (d) exhibited reduced apoptosis. TUNEL staining is shown in green and red; blue represents the counterstain.

As these findings suggest that BMP4/p38/caspase-3 signaling may play a key role in establishing podocyte injury, we investigated the involvement of these molecules *in vivo*. Cleaved caspase-3 levels were increased in the glomeruli of STZ-induced DN mice (Fig. 6A-c). The area expressing cleaved caspase-3 overlapped with both mesangial cells and podocytes. STZ-induced *Bmp4* knockout mice exhibited reduced levels of cleaved caspase-3 (Fig. 6A-c and d), whereas increased levels of cleaved caspase-3 were detected in the tamoxifen-induced *Bmp4* and *CAG-B4* × *Pod-Cre* mice (Fig. 6A-f and h). Diabetic-induced nuclear damage in wild-type mice was reflected by increased apoptosis, as highlighted by TUNEL staining. The diabetic *Bmp4* +/− mice exhibited notably reduced apoptotic changes, as assessed by TUNEL staining (Fig. 6B-c and d). *Bmp4* tgm (+) and *CAG-B4* × *Pod-Cre* mice displayed nuclear damage due to apoptosis (Fig. 6B-j red color and l green color). The green color in images of *Bmp4* tgm is GFP, which is derived from the recombinant gene.

## Discussion

Podocyte injury and loss have been indicated as the crucial markers of the pathogenesis of glomerular injury; however, the detailed molecular mechanisms and cell-to-cell responses remain to be elucidated, because multiple factors may cause podocyte injury. In the glomerulus, three types of cells (i.e., endothelial, mesangial, and parietal epithelial) respond to podocyte injury. Endothelial and mesangial cells are connected to podocyte cells across the glomerular basement membrane. However, the detailed mechanisms underlying the relation between the mesangium and podocyte injury are unclear. DN is characterized by mesangial matrix expansion, which is caused by the excess deposition of extracellular matrix proteins in the mesangial area, as detected by increased expression of type IV collagen. The initial finding of the present study was that BMP4 secreted by glomerular cells, including mesangial cells and podocytes, directly caused podocyte injury and correlated with urinary albumin excretion. BMP4 secreted from cells adheres to collagen IV^22^. We postulate that BMP4 secreted from podocytes accumulates in glomerular mesangial cells, in which collagen IV is a primary component. Concomitantly, BMP4 accumulates in mesangial cells, activates Smad1, and increases the production of extracellular matrix proteins in mesangial cells. BMP4 expression was primarily observed in mesangial cells, as well as in podocytes. BMP4 is a secreted protein that may act on various cells in a paracrine manner. Therefore, BMP4 signaling pathways play central roles in the development of podocyte injury and mesangial matrix expansion in subjects with DN.

P38 MAPK signaling mediates BMP4-induced apoptosis and senescence in cancer cells^23^, whereas BMP4-induced Smad1 signaling activates apoptotic cell death in embryonic stem cells^24^. Although the p38 MAPK and Smad1 pathways have been reported to synergistically induce apoptosis and autophagy in tumor cells^25^, other studies have reported that the p38 MAPK pathway is separate from Smad1 signaling because it mediates NRAGE-p38 signaling^26^. In particular, the mechanism underlying podocyte damage depends on BMP4/BMP receptor signaling in the early stage of diabetes. BMP4 induced podocyte apoptosis mediated by p38 activation and cleaved caspase-3 (Fig. 5B). This pathway induced podocyte loss, as detected by the number of WT1-positive cells, and subsequent foot process injury, as evidenced by nephrin staining (Fig. 4A-h,j and n).

R-Smad has been reported to affect the cell cycle; accordingly, Smad1 may impact senescence in podocytes^27^. Alternatively, we considered that Smad1 upregulated the extracellular matrix expansion in mesangial cells, based on our observation that phosphorylated Smad1 directly induces collagen IV expression in mesangial cells^6^. The Smad1 inhibitor did not affect the levels of cleaved caspase-3 in cultured podocytes. However, the induction of apoptosis was suppressed by p38 inhibitors (Fig. 5C). BMP4 exerts two direct effects on glomeruli injury, including the effect of BMP4/Smad1 signaling on mesangial matrix expansion and the effect of BMP4/p38 signaling on podocyte injury. As shown in our previous study, a BMP4 neutralizing antibody blocked Smad1-mediated mesangial matrix expansion^21^. Based on these findings, BMP4 signaling represents a promising target for developing a therapeutic approach for DN.

Podocyte-specific *Bmp4* tgm showed typical mesangial sclerosis accompanied by podocyte loss and increased phosphorylated Smad1 levels in mesangial cells. Thus, podocyte loss itself is responsible for mesangial sclerosis, and the increased BMP4 expression observed in podocytes induces Smad1 activation in mesangial cells in a paracrine fashion. Based on these findings, BMP4/p38 signaling in podocytes and BMP4/Smad1 signaling in mesangial cells are potential therapeutic targets for DN. Because BMP4 regulates both podocyte loss and mesangial Smad1 activation in DN, BMP4 represents an essential molecule involved in the development of the typical pathological features of DN.

Both podocyte loss and mesangial expansion were concomitantly observed in most pathological cases of DN; however, microalbuminuria did not completely reflect the characteristic renal pathological changes, including mesangial matrix expansion and glomerular basement membrane thickening, in the early stages of human DN. Urinary albumin excretion is affected by the charge and size of the barrier of the glomerular basement membrane^28,29^. The mechanism of renal disease progression requires a subsequent in-depth analysis of the relationship between podocyte dysfunction and GBM disruption. *Bmp4* knockout mice exhibited reduced podocyte injury and albuminuria levels. These initial observations provide sufficient evidence that BMP4 is a therapeutic target. Based on the findings of the present study, we speculate that BMP4 induces podocyte injury in an early stage of DN in which microalbuminuria is observed. Additional studies are necessary to confirm these observations.

## Experimental Procedures

### Experimental animals

All animal experiments were performed in accordance with the guidelines of the Institutional Animal Care and Use Committee of Chugai Pharmaceutical Co., Ltd. and the Tokushima University animal facility, and the Ethical Review Board of Tokushima University granted permission for the procedures performed in this study.

*Bmp4*-knockout mice (C57BL/6 *Bmp4*+/−) and conditional knockout mice (C57BL/6 *Bmp4*^*loxP*^) were gifts from B. Hogan and M. Saitou. The generation and characterization of these mutant mice have been described elsewhere^16,30^. The *Bmp4* +/− mice were maintained on a C57BL/6 background. The *Podocin-Cre* (*Pod-Cre*) mice were obtained from The Jackson Laboratory (Bar Harbor, ME, USA). We crossed *Pod-Cre* mice with *Bmp4*^*loxP*^ mice to generate the podocyte-specific *Bmp4* knockout mice. Diabetes was induced in 8-week-old male C57BL/6 mice weighing 22–24 g by the intraperitoneal injection of 50 mg per kg body weight streptozotocin (STZ) in citrate buffer, pH 4.5, for 5 consecutive days. The diabetic state was confirmed 4 weeks after the final injection by measuring the blood glucose levels with an Accu-Chek monitor (Roche). All mice that were administered STZ had a blood glucose concentration exceeding 400 mg/dl and were considered diabetic. Reference mice were injected with citrate buffer. The mice were classified into four groups: (A) nondiabetic C57BL/6 (n = 7), (B) nondiabetic *Bmp4* +/− (n = 7), (C) diabetic C57BL/6 (n = 9), and (D) diabetic *Bmp4* +/− (n = 9) mice. These mice were euthanized 24 weeks after the final injection of STZ and citrate buffer. Glycoalbumin levels were measured using an enzyme assay (Lucica GA-L, Asahi Kasei Co., Ltd.). Microalbuminuria was measured using a DCA vantage analyzer (SIEMENS Healthineers). The creatinine concentration was measured with an enzyme assay (Serotec Co., Ltd.). The procedure for generating tamoxifen-inducible *Bmp4* tgm is described in a previous report^7^. Eight- to 10-week-old transgenic mice were fed a diet (CE-2, CLEA Japan) containing 0.02% tamoxifen citrate (Sigma) to induce the expression of the *Bmp4* gene. Control mice were fed a normal diet (CE-2). The *CAG-CAT-Bmp4* transgenic mice (*CAG-B4*) were provided by RIKEN Bio Resource Center (Tsukuba, Japan) under an agreement with Dr. Y. Saga (National Institute of Genetics, Japan). We generated the double tgm by mating *CAG-B4* mice with *Pod-Cre* mice. *CAG-B4 x Pod-Cre* mice overexpressed BMP4 in podocytes. Reference mice were not mated with *Pod-Cre mice*. These mice were monitored for 1 year. Blood pressure levels were measured with a non-invasive blood pressure device (BP-98A-L, Softron Co., Ltd.) attached to the animal’s tail.

### Morphology studies

Tissues used for light microscopy studies were fixed with 10% neutral buffered formalin. One-μm-thick sections were prepared from paraffin-embedded tissues and subjected to periodic acid-Schiff (PAS), periodic acid methenamine silver (PAM) and picrosirius red staining. Morphometric analyses of PAM-stained tissues were performed using ImageJ software. The glomerular surface area and the PAM-positive area/glomerular area (%) were measured for 30 glomeruli in each mouse.

The avidin-biotin complex (ABC) method was employed for immunohistochemistry to detect the expression of various proteins. Tissue sections were deparaffinized, rehydrated with PBS and treated with 3% H_2_O_2_. Subsequently, tissue sections were subjected to an antigen retrieval procedure in HistoVT one buffer according to the manufacturer’s instructions (Nacalai Tesque, Inc., Japan). Sections were incubated with various primary antibodies. Briefly, 4-μm-thick cryostat sections were prepared, air-dried for an hour and fixed with 4% paraformaldehyde for 20 min at 4 °C. After equilibrating the sections with PBS, they were incubated with various primary antibodies overnight at 4 °C, washed with PBS and then incubated with FITC-conjugated secondary antibodies conjugated (Thermo Fisher Scientific) for 1 hr. Following a PBS wash, tissue sections were covered with a drop of buffered glycerol, mounted with a cover slip and examined with a fluorescence microscope (KEYENCE CORPORATION). Anti-BMP4 (Abcam), anti-WT1 (Santa Cruz Biotechnology), anti-nephrin (IBL), anti-cleaved caspase-3 (Cell Signaling Technology) antibodies were used for these procedures. Nephrin immunoreactivity was quantified by calculating the nephrin-positive areas in the glomeruli using ImageJ software. The WT1-positive cells were counted in the glomeruli.

Tissues used for electron microscopy were fixed with 2.5% glutaraldehyde. We entrusted electron microscopy analysis to a specialized company (BML, Inc.).

### Cell culture experiment

Conditionally immortalized murine podocytes were obtained from a cell line service (Eppelheim, Germany). Podocytes were cultured in RPMI-1640 medium (Sigma) containing 10% fetal calf serum, 100 U/ml penicillin, 100 mg/ml streptomycin (Invitrogen) and 2 mM L-glutamine at 33 °C in a 5% CO_2_ atmosphere. Cells were cultured on type I collagen-coated dishes at 37 °C for 1 week to induce differentiation. After differentiation, cells were starved for 24 h in Opti-MEM (Invitrogen), and then incubated with BMP4 (R&D Systems) at 37 °C for the indicated time points. For the inhibition assay, cells were treated with dorsomorphin (Smad1 inhibitor), SB202190, SB242235 or SB203580 (p38 inhibitors) and stimulated with BMP4.

### Immunoblot analyses

Cell extracts were prepared from podocytes, as previously described. Protein concentrations in the extracts were measured using the Bradford method, and equal amounts of protein were loaded in each lane of gels, subjected to SDS-PAGE and blotted onto PVDF membranes, which were incubated with a 1 μg/ml dilution of each of the primary antibodies overnight at 4 °C. After a brief wash with TBS-T, membranes were then incubated with a diluted HRP-conjugated secondary antibody (Thermo Fisher Scientific). Autoradiograms were prepared using an enhanced chemiluminescence (ECL) detection system (Thermo Fisher Scientific). Equal loading of the samples was confirmed by probing the immunoblots with β-actin, α-tubulin or GAPDH antibodies. Anti-pSmad1/5/9 (Cell Signaling Technology), anti-pp38 (Cell Signaling Technology), anti-cleaved caspase-3 (Cell Signaling Technology), anti-podocin (IBL), anti-β-actin (Sigma), anti-α-tubulin (SIGMA), and anti-GAPDH (Abcam) antibodies were used for these procedures. A TUNEL assay was used to detect apoptosis (Millipore).

### Statistical analyses

The results of the statistical analyses are reported as the means ± S.E. Student’s t-test or ANOVA was used to compare the data between each group. Albuminuria was statistically analyzed using a nonparametric method. GraphPad Prism 7 software was used for all statistical analyses. *P* values of less than 0.05 were considered statistically significant.

## Electronic supplementary material

Supplementary Information

## Data Availability

The tamoxifen-inducible *Bmp4* transgenic mice are unavailable to the readers because of the licensing agreement between Chugai Pharmaceutical Co., Ltd. and the third party regarding a gene introduced to the mice.
