# Supplementary material for: An adjustment in BMP4 function represents a treatment for diabetic nephropathy and podocyte injury
Source: Sci Rep. 2018 Aug 29;8:13011. doi: 10.1038/s41598-018-31464-9 (PMC6115362; doi:10.1038/s41598-018-31464-9)
Supplement: Supplementary file 1 — Supplementary Information [file 41598_2018_31464_MOESM1_ESM.docx]

**Supplementary Information**

**An adjustment in BMP4 function represents a treatment for diabetic nephropathy and podocyte injury**

Yui Fujita, ^＊1^ Tatsuya Tominaga, ^＊1^ Hideharu Abe, ^1^ Yumi Kangawa, ^1^ Naoshi Fukushima,^2^ Otoya Ueda,^2^ Kou-ichi Jishage, ^2, 3^ Seiji Kishi, ^1^ Taichi Murakami, ^1^ Yumiko Saga,^4^ Yashpal S Kanwar,^5^ Kojiro Nagai, ^1^ Toshio Doi^1^

1. Department of Nephrology, Graduate School of Biomedical Science, Tokushima University, Tokushima, Japan. 2. Research Division, Fuji Gotemba Research Labs, Chugai Pharmaceutical Co., Ltd., Shizuoka, Japan. 3. Chugai Research Institute for Medical Science Inc., Shizuoka, Japan. 4. Division of Mammalian Development, Genetic Strains Research Center, National Institute of Genetics, Mishima, Shizuoka, Japan. 5. Department of Pathology & Medicine-Nephrology, FSM, Northwestern University.

*Correspondence should be addressed to Tatsuya Tominaga, Ph. D.

Graduate School of Biomedical Science, Tokushima University, Tokushima, 770-8503, Japan

Tel: 81-88-633-9070, Fax: 81-88-633-9063,

E-mail: [tominaga.tatsuya@tokushima-u.ac.jp](mailto:tominaga.tatsuya@tokushima-u.ac.jp)

List of Materials

Supplementary Table 1

Supplementary Figure 1
